# Closing Human Reference Genome Gaps: Identifying and Characterizing Gap-Closing Sequences

**DOI:** 10.1534/g3.120.401280

**Published:** 2020-06-12

**Authors:** Tingting Zhao, Zhongqu Duan, Georgi Z. Genchev, Hui Lu

**Affiliations:** * SJTU-Yale Joint Center for Biostatistics, Department of Bioinformatics and Biostatistics, School of Life Sciences and Biotechnology, Shanghai Jiao Tong University, Shanghai, China; ^†^ Shanghai Engineering Research Center for Big Data in Pediatric Precision Medicine, Center for Biomedical Informatics, Children’s Hospital, Shanghai, China; ^‡^ Bulgarian Institute for Genomics and Precision Medicine, Sofia, Bulgaria

**Keywords:** genomic gaps, human genome, de novo assemblies, gap closure, non-reference sequences

## Abstract

Despite continuous updates of the human reference genome, there are still hundreds of unresolved gaps which account for about 5% of the total sequence length. Given the availability of whole genome *de novo* assemblies, especially those derived from long-read sequencing data, gap-closing sequences can be determined. By comparing 17 *de novo* long-read sequencing assemblies with the human reference genome, we identified a total of 1,125 gap-closing sequences for 132 (16.9% of 783) gaps and added up to 2.2 Mb novel sequences to the human reference genome. More than 90% of the non-redundant sequences could be verified by unmapped reads from the Simons Genome Diversity Project dataset. In addition, 15.6% of the non-reference sequences were found in at least one of four non-human primate genomes. We further demonstrated that the non-redundant sequences had high content of simple repeats and satellite sequences. Moreover, 43 (32.6%) of the 132 closed gaps were shown to be polymorphic; such sequences may play an important biological role and can be useful in the investigation of human genetic diversity.

The human reference genome, first delivered by the Human Genome Project in 2001 (Lander *et al.* 2001; Venter *et al.* 2001), is an invaluable scientific roadmap widely used in biomedical studies and genetic research (International Human Genome Sequencing Consortium 2004). However, nearly 20 years later, even the most current version (GRCh38), still has a multitude of unsolved genomic gaps (>150 Mb, about 5% of the human genome sequence) (Schneider *et al.* 2017). The continuous advancement of sequencing technologies, now in their third-generation, has enabled the sequencing and assembling of individual genomes (Chaisson *et al.* 2015; Pendleton *et al.* 2015; Shi *et al.* 2016; Li *et al.* 2010; Jain *et al.* 2018; Audano *et al.* 2019; Huddleston *et al.* 2017). The emergence of these new datasets provides the opportunity to bring new insights into the remaining unmapped genomic dark matter and close the genomic gaps (Sedlazeck *et al.* 2018).

Previous studies have contributed to solving this problem and several genomic sequences were shown to fit in the gap regions in the current version of the human genome assembly. In a recent (2015) study, a haploid human genome (CHM1) was obtained by single-molecule real-time (SMRT) sequencing and 31 of 172 interstitial euchromatic gaps within GRCh38 were closed through a local assembly approach, resulting in the addition of 40 kb sequences to the reference genome (Chaisson *et al.* 2015). In the same year, another study used the NA12878 *de novo* assembly performed by SMRT sequencing and closed 28 interstitial gaps in GRCh38 with 34 kb of assembled sequences (Pendleton *et al.* 2015). Similarly, the *de novo* assembly of a Chinese individual (HX1) was used in a 2016 study to completely close 37 gaps with 222 kb sequences of continuous N-marked runs on GRCh38 primary assembly sequences through mapping flanking sequences upstream and downstream of the gaps to HX1 (Shi *et al.* 2016). A further study in the same year utilizing the AK1 *de novo* assembly closed 105 euchromatic gaps and a total of 364 kb sequences were added to the reference genome (Seo *et al.* 2016). In a 2018 work, *de novo*–assembled contigs of GM12878 helped identify sequences that close 12 gaps in GRCh38, each new sequence was longer than 50 kb, in total summing to 83 kb of previously unknown euchromatic sequences (Jain *et al.* 2018).

Identifying and understanding sequences that fill the open genomic gaps could provide more comprehensive perspectives on the complexities in the human genome. Initial characterization of the gap-closing sequences discovered by the aforementioned studies shows that simple tandem repeats and satellite sequences are significantly enriched within the closed gaps (Pendleton *et al.* 2015; Seo *et al.* 2016; Chaisson *et al.* 2015; Shi *et al.* 2016). Additionally, reference genome gap regions may contain functional genomic elements (Jain *et al.* 2018). Such recent studies have made significant progress regarding genomic gaps, however unanswered questions still remain. Almost all the existing studies were focused on using single individual genomes and emphasized the description and closure of interstitial euchromatic gaps. In this work, we employ multiple whole genome *de novo* assemblies to systematically identify and characterize gap-closing sequences for all unsolved types of gap regions in the human reference genome.

## Materials And Methods

### Methods and workflow summary

**Figure 1:**
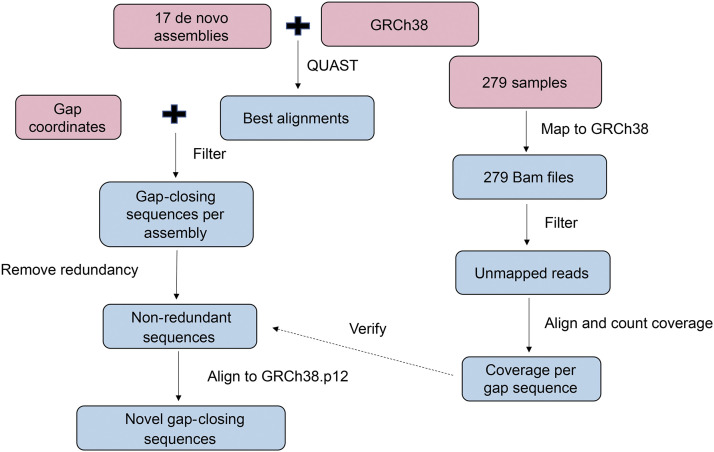
Workflow overview of the identification and validation of gap-closing sequences. Pink blocks represent inputs and blue blocks represent intermediate and final results.

Our methodology (Figure 1) utilized several genomic datasets including the current version of the human reference (GRCh38.p12), 17 human *de novo* assemblies, sequencing data from Simons Genome Diversity Project (SGDP) (Mallick *et al.* 2016), 4 non-human primate genomes and non-references sequences (NRS) from other studies (Sherman *et al.* 2019; Li *et al.* 2019; Audano *et al.* 2019). The gaps in GRCh38 were first classified as euchromatic and non-euchromatic according to their coordinates. Next, *de novo* assemblies from 17 human individuals and assembly-to-assembly alignment were used to determine GRCh38 gap-closing sequences. We then clustered them to remove the redundant sequences among different individual genomes. At the completion of this step, the gap-closing sequences were identified. Furthermore, we confirmed whether these gap-closing sequences could be found in other datasets. Finally, we performed annotation of the discovered sequences. Details regarding the methodology workflow, genomic data, and software programs utilized follows below.

### Genomic data

#### Human de novo assemblies:

Seventeen human *de novo* assemblies which were sequenced by the long-read SMRT PacBio RS II and Sequel sequencing platforms were downloaded from the NCBI (ftp://ftp.ncbi.nlm.nih.gov/genomes/genbank/vertebrate_mammalian/Homo_sapiens/latest_assembly_versions/) (Table S1). The 17 assemblies covered major human ethnic groups and consisted of the following: five Africans (HG02818, HG03486, NA19240, NA19434 and YRI), five East Asians (AK1, HG00514, HG02059, HX1 and ASM185674v1), four Europeans (CHM13, NA12878, ASM101398v1 and NA24385), two Americans (HG00733 and HG01352), and one South Asian (HG03807).

#### Reference genome:

The GRCh38.p12 of the human genome reference, including primary assembly sequences (22 autosomes, 2 sex chromosomes and mitochondria), alternative loci and patch sequences, were downloaded from ftp://hgdownload.soe.ucsc.edu/goldenPath/hg38/bigZips/latest/hg38.fa.gz.

#### Non-human primate genomic data:

The reference genome sequences of four non-human primates: *chimpanzee* (GCA_002880755.3), *bonobo* (GCF_000258655.2), *gorilla* (GCA_900006655.3), and *orangutan* (GCF_002880775.3) were downloaded from NCBI websites according to the accession numbers.

Simons Genome Diversity Project (SGDP) dataset: The deep whole genome sequencing data from Illumina HiSeq 2000 platform of 279 individuals was downloaded from the European Bioinformatics Institute (EBI) (accession PRJEB9586). This data were obtained by the SGDP which reported multiple high-quality individual genomes across 142 diverse populations (Mallick *et al.* 2016). Detailed information of the samples is described in Table S2.

#### Three published NRS datasets:

The first dataset was African pan-genome (APG) contigs assembled from 910 African-descent individuals (Sherman *et al.* 2019), which included 296.5 Mb sequences in 125,715 distinct contigs (GenBank accession code PDBU01). The second dataset consisted of 129.1 Mb NRS derived from 31 human *de novo* assemblies and the sequences were extracted according to sequence information provided by Li *et al.* (NRS_Li) (Li *et al.* 2019). The last dataset consisted of major structural variants (MSVs) including insertions, deletions, and inversions of 50 bp or greater relative to GRCh38 from fifteen long-read *de novo* human genomes (Audano *et al.* 2019).

### Gap-closing sequences discovery

Euchromatic and non-euchromatic gaps definition: In total, there are 783 unclosed gaps (annotated with Ns) distributed across 150 Mb genomic regions of the GRCh38 primary assembly sequences, which are categorized as “Within_scaffold” and “between_scaffolds”, “heterochromatin”, “short_arm”, and “telomere” (Table S3). Here, the term “within-scaffold” gaps refers to the case when sequences on either side of the gap are in a single scaffold and “between_scaffolds” gaps refers to the case when sequences on either side of the gap are in separate scaffolds. We screened for euchromatic gaps following a previous strategy (Seo *et al.* 2016). For the euchromatic gaps, we only included “within_scaffold” and “between_scaffolds” gaps and filtered out gaps located in modeled regions. Gaps adjacent to modeled regions and positioned within the 5 kb flanking sequences from those classified as ”telomere”, ”heterochromatin”, or ”short_arm” were also removed; the gaps lying in ”acen”, and ”gvar” (acrocentric p-arms of chr21 and 22) and ”stalk” were excluded from the set of euchromatic gaps. Reference genome modeled regions and gap location information were obtained from the NCBI at https://www.ncbi.nlm.nih.gov/projects/genome/assembly/grc/human/data/38/Modeled_regions_for_GRCh38.tsv and ftp://ftp.ncbi.nlm.nih.gov/genomes/all/GCA/000/001/405/GCA_000001405.15_GRCh38/GCA_000001405.15_GRCh38_genomic.gaps.gz. As a result of this step, 191 gaps were classified as euchromatic and the remaining 592 were classified as non-euchromatic gaps (Table S4).

#### Pair-wise alignments between the human reference genome and 17 human de novo assemblies:

We used the assembly-to-assembly method applied to the human reference genome and the 17 human *de novo* assemblies; non-overlapping best alignment set of chains provided by QUAST (version 5.0.2) (Mikheenko *et al.* 2018) flanking or spanning a gap region were used as candidates to close the gaps. All the 17 assembled genomes were aligned to the GRCh38 primary assembly sequences by minimap2 (Li 2018) implemented in QUAST with the following parameters:”-c -x asm5 –mask-level 0.9 –min-occ 200 -g 2500 –score-N 2 –cs -t 8”. For comparison, we also adopted a more precise but slower aligner MUMmer (version 4.0.0beta2) (Kurtz *et al.* 2004).

#### Candidate set evaluation and sequence selection:

We examined the alignment record whether the spanning of one scaffold’s best alignment included any gaps. When two alignments with consistent orientation aligned to the same contig were located on either side of one gap and the distances from the gap edges were less than 100 bp, we subtracted the sequence between the two alignments of the scaffold and considered it as a gap-closing sequence. Alternatively, if a continuous alignment spanned the gap region, flanking sequences upstream and downstream of the gaps in the aligned sequence of GCRh38 were mapped to the aligned sequence of the *de novo* assemblies with the software minimap2 (Li 2018) (parameters:-c -x asm5 –mask-level 0.9 –min-occ 200 -g 100–score-N 2 –cs -t 8) and the unaligned part of the scaffold represented the looked-for gap-closing sequence.

#### Sequence clustering and redundancy removal:

We merged all the gap-closing sequences obtained from all 17 genomes and clustered sequences located at the same gap to generate the non-redundant call set by CD-HIT (Fu *et al.* 2012) with identity threshold of 90% and length difference cutoff of 90% (-c 0.9 -s 0.9). Shared sequences in every assembly were also obtained by considering the clustering results.

### Analysis

The analysis step included validation of the gap-closing sequences, confirming their presence in other genomic datasets, and finally - annotation of the sequences.

#### Finding unmapped reads and calculating coverage:

We checked whether the non-redundant sequences located in gap regions could be captured by short-reads from Illumina platform in other datasets. First, the sequencing data of 279 individuals from the SGDP dataset were mapped to the GRCh38 primary assembly sequences using Bowtie2 (Langmead and Salzberg 2012) with default parameters. All the unmapped reads (including paired-end unmapped reads and single-end unmapped reads) were extracted with SAMTools (Li *et al.* 2009; Li 2011). We then remapped the unmapped reads to the non-redundant gap-closing sequences, with 99 bp flanking sequences on both sides. Finally, breadth of coverage (percentage of bases in sequences that could be covered with given number of reads) and depth of coverage (average number of reads covered all bases of sequences) of the gap-closing sequences were calculated by SAMTools with related custom scripts.

#### Presence of the gap-closing sequences in other datasets:

We examined the presence of the gap-closing sequences in GRCh38.p12, four non-human primate genomes, APG contigs, NRS_Li, and MSVs by aligning gap-closing sequences to them using the nucmer program implemented in the software MUMmer (-c 100 -l 100 -maxmatch). The criteria for presence of the sequences was determined by >= 95% identity and >= 80% coverage.

#### Annotation of the repeats in gap-closing sequences:

The repeat elements of gap-closing sequences were annotated by RepeatMasker (Jurka 2000), which is a program that screens DNA sequences for interspersed repeats and low complexity DNA sequences. CpGProD (Ponger and Mouchiroud 2002) were used to identity CpG islands.

### Data availability

Related computer scripts used to discover gap-closing sequences and analysis are available at https://github.com/ranluo7/gap-closure. Supplemental material available at figshare: https://doi.org/10.25387/g3.12206744.

## Results

### Gap-closing sequences discovery

**Figure 2:**
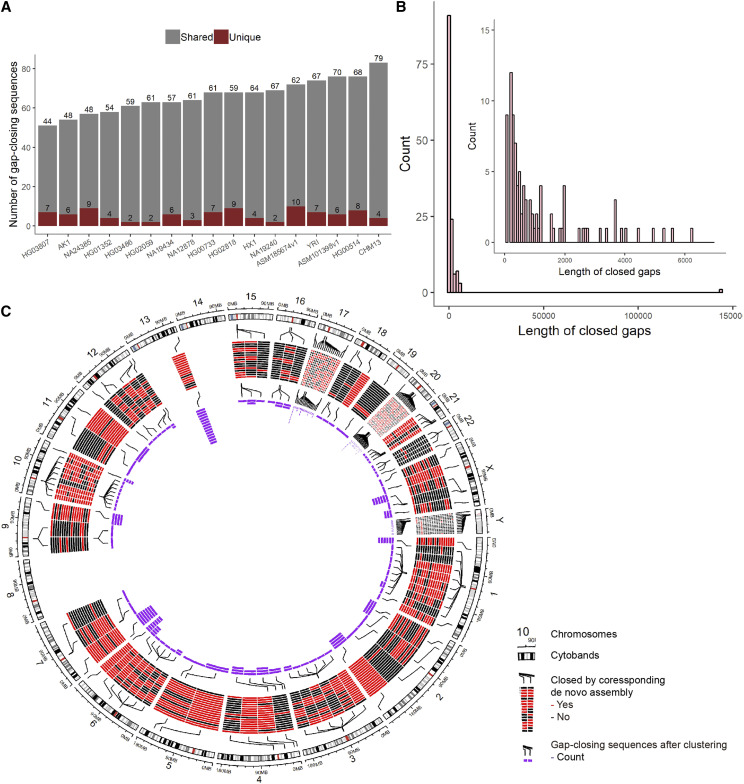
Discovered gap-closing sequences. (A) Number of the gap-closing sequences in each *de novo* assembly. Gray bars represent gap-closing sequences also found in other assemblies, red bars represent individual-specific gap-closing sequences. (B) Length distribution of the gaps we closed. As most of gaps were closed by multiple assemblies, the length of gaps is defined as the median length of gap-closing sequences from the different assemblies. (C) A Circos plot showed all gaps we closed and the number of gap-closing sequences before and after clustering. The first track represents GRCh38 and its cytobands. The second track is a heatmap indicating if the gap was closed by one of 17 *de novo* assemblies and there are connection lines connecting heatmaps and original positions in the genome. The third track represent the number of gap sequences after removal of redundancy.

In total, we identified 1,125 gap-closing sequences (Table S5) covering 132 genomic gaps in the 17 human *de novo* genomes utilized in this study. On average, 66 gap-closing sequences were found per each *de novo* genome and 91.5% of the 1,125 gap-closing sequences were also found in other genomes with >= 90% identity and >= 90% coverage (Figure 2A). The average and median length of the gaps we closed were 1.9 kb and 300 bp (Figure 2B). Within the set of the 1,125 gap closing sequences, most sequences that were associated with the same gap could be clustered (Figure 2C), indicating consistency of the gap-closing sequences across multiple genomes. Interestingly, there were 15 gaps which seem to point to a discrepancy in the reference genome. These gaps reveal situations in which no sequences are actually missing in the reference or in which there is an apparent overlap between the flank sequences of these “gaps”. For example, gap_174 (chr5:155760324-155761324) seemed to be complete (*i.e.*, it is not a gap) which appeared the same in all 17 *de novo* assemblies and was consistent with previous studies (Seo *et al.* 2016). These potentially erroneous gap annotations in the published genomes may arise from contig mis-assembly, structural polymorphisms, expansion of repetitive elements, or errors when tiling contigs into a reference (Audano *et al.* 2019). After removing redundant sequences, we obtained a call set of 212 non-redundant gap-closing sequences (Table S6) distributed across 117 gaps which summed up to 2.3 Mb with a median length of 697 bp. Among them, several gaps with more than one non-redundant gap-closing sequence were defined as polymorphic and 43 (32.6%) of the 132 gaps showed as polymorphic. Each divergent sequence could be supported by an average of more than three assemblies (range from 1 to 15), which showed the robustness of these divergent sequences. Moreover, we compared results using another aligner (MUMmer) on one individual (NA24385). Results suggested that most closed gaps identified by minimap2 and MUMmer were identical and the discovered gap-closing sequences corresponding to those gaps were also consistent (Table S7, Figure S1), indicating the feasibility of our method.

### Validation of gap-closing sequences by coverage calculation

**Figure 3:**
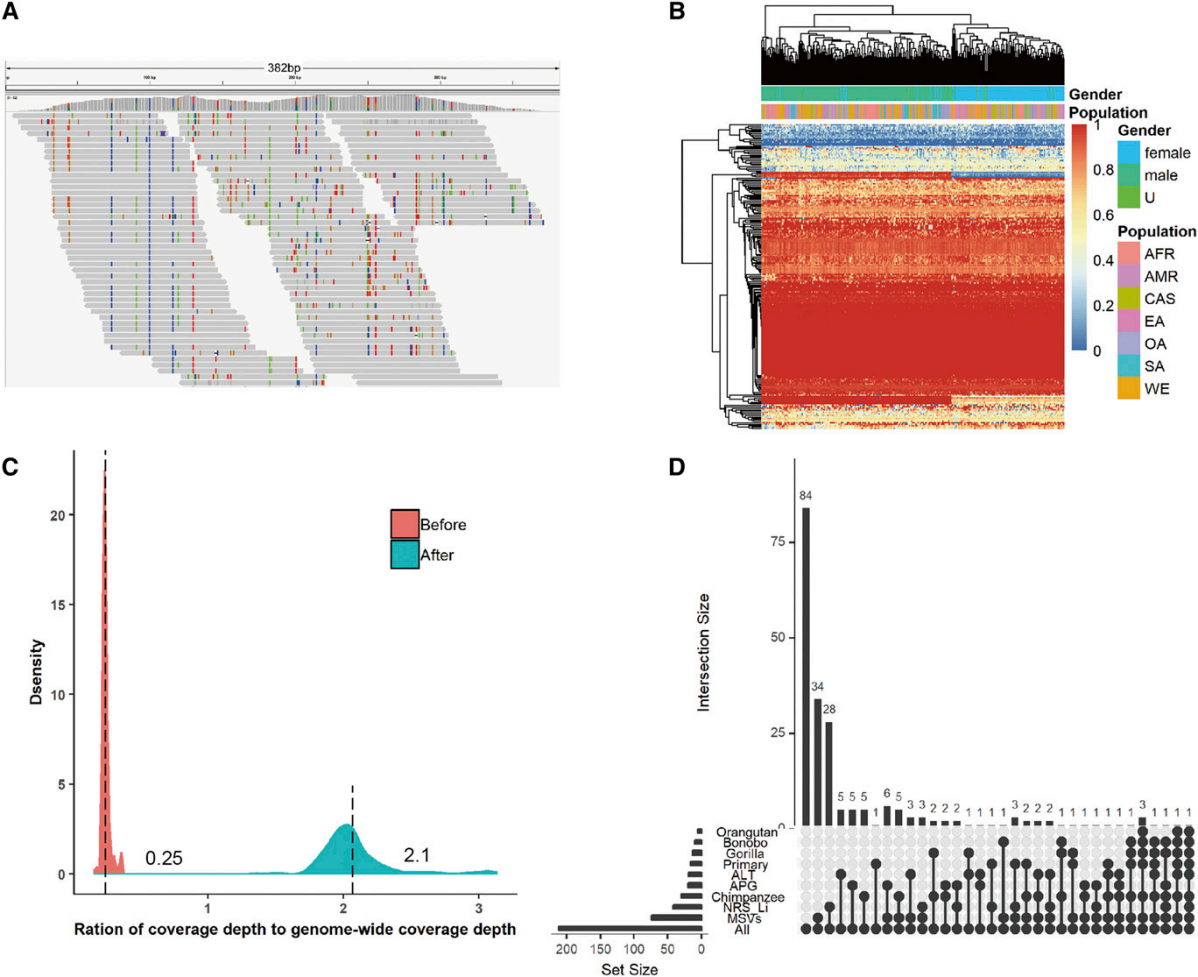
Validation of non-redundant gap-closing sequences.(A) IGV (Robinson *et al.* 2011; Thorvaldsdóttir *et al.* 2012) illustrating an example of a gap (Gap_564, chr20:28820603-28820663) closed by 184 bp sequences from the *de novo* assembly (NA12878). Gap-closing sequence with both 99 bp flanking sequences could be completely covered by unmapped reads from the sample ERR1625860. (B) Heatmap showing breadth of coverage information of 212 non-redundant sequences by unmapped reads from 279 samples. Breadth of coverage is the percentage of bases in sequences that are covered with a certain depth and here was calculated as the percentage of sites in sequences at 1x depth. Horizontal axis represents 279 samples. Vertical axis represents 212 non-redundant gap-closing sequences. (C) Density plot of the ratio of coverage depth to genome-wide coverage depth. Depth here refers to the average number of times that sites in sequences are mapped by reads and genome-wide coverage depth refers to sequencing depth of SGDP (Table S2). Ratio of coverage depth to genome-wide coverage depth is coverage depth of gap-closing sequences divided by the sequencing depth. ”Before” here refers to coverage depth of gap-closing sequences and ”After” means combining the coverage depth of divergent sequences for the same gap, which is calculated as the sum of coverage depth of divergent sequences for the same gap divided by the maximum length of these divergent sequences. (D) Intersection of gap-closing sequences present in GRCh38 primary assembly sequences (Primary), alternative loci and patch sequences (ALT), four non-human primates, APG contigs, NRS_Li, and MSVs.

We calculated the breadth of coverage of the non-redundant gap-closing sequences by the unmapped reads in 279 individual genomes (SGDP dataset). Some gap-closing sequences could be completely covered by unmapped reads (Figure 3A). Overall breadth of coverage information of 212 non-redundant gap-closing sequences by unmapped reads from the 279 samples can be seen on the heatmap in (Figure 3B). When coverage more than 80% was defined as a cutoff, 91.0% of the non-redundant sequences were covered at 1x depth by at least one sample. Due to the fact that female individual genomes lack chromosome Y, the 279 samples could be grouped into two classes relating with gender based on breadth of coverage of sequences. After removing gap-closing sequences located in chromosome Y, this situation disappeared (Figure S2A). Moreover, we examined the depth of coverage of these gap-closing sequencing compared with the general depth of the genome in the 279 samples (Figure 3C). We found that coverage depth of gap-closing sequences is only 25% (mean = 0.25, sd = 0.03) of the genome-wide coverage depth, partly because unmapped reads of the samples underestimate the number of reads mapping to gap-closing sequences, repeats, and polymorphisms enriched in gap-closing sequences. Thus, we also calculated the coverage depth in units of gaps by combining the coverage depth of divergent sequences for the same gap. The coverage depth after combining the polymorphic sequences increased significantly. Furthermore, we found that several gap-closing sequences showed a population-specific pattern (Fisher test *P* < 0.001, Figure S2B).

### Aligning non-redundant gap closing sequences to the reference genome

Of the discovered (n = 212) non-redundant gap-closing sequences, 31 (14.7%) sequences could be aligned (identity >= 95%, coverage >= 80%) to GRCh38.p12 and 19 of 31 sequences could only be aligned to the patch or alternate sequences of GRCh38 (Figure 3D). The 31 sequences added up to only 23 kb with a median 377 bp and accounted for a small portion of the total length of the 211 non-redundant sequences (2.3 Mb). Alignment of the 19 sequences to the patched references is an expected result, however the fact that the remaining 12 sequences aligned to the GRCh38 primary assembly sequences may be explained by the presence of extra copies of small repetitive elements in these short sequences, and by considering that most of them (9/12) have nearly 100% repetitive bases. The remaining 181 non-redundant gap-closing sequences added 2.2 Mb NRS to the human reference genome.

### Presence of discovered gap-closing sequences in non-human primate genomes

To determine the origin of 212 non-redundant gap-closing sequences, we aligned them to four different non-human primate genomes. Several gap-closing sequences could be aligned to the chimpanzee (30, 14.2%), the gorilla (13, 6.1%), the bonobo (10, 4.7%), and the orangutan (5, 2.4%) genome (Figure 3D). This trend correlates well with the evolutionary relationship between Homo sapiens with these non-human primates which is in accordance with previous studies (Prüefer *et al.* 2012; Chimpanzee Sequencing and Analysis Consortium *et al.* 2005; Scally *et al.* 2012; Wong *et al.* 2018). In aggregate, 33 (15.6%) non-redundant gap-closing sequences were found in non-human primate genomes; of these sequences, 18 gap-closing sequences were not aligned to the human reference genome. These gap-closing sequences had high breadth of coverage (mean = 0.9, sd = 0.18) by reads from the SGDP samples, suggesting that they are ancestral to humans.

### Presence of discovered NRS in other NRS datasets

We compared our discovered NRS with three previously published results (Sherman *et al.* 2019; Li *et al.* 2019; Audano *et al.* 2019) which reported a list of NRS (see materials and methods). In summary, 18 (9.9%) gap-closing NRS were present in APG contigs, 35 (19.3%) were present in NRS_Li and 55 (30.4%) were present in MSVs (Figure 3D). The remaining 84 gap-closing NRS suggested that our discovered NRS could be complementary to previously reported NRS.

### Characterization of gap-closing sequences

**Figure 4:**
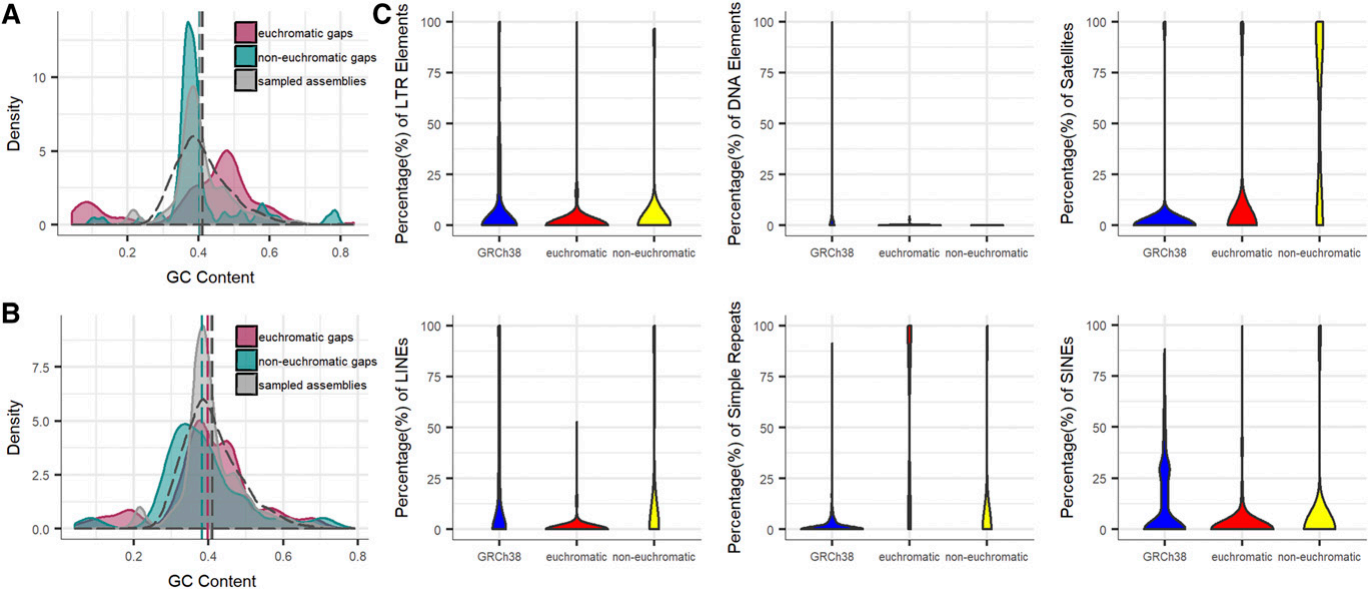
Characterization of gap-closing sequences. (A) Density plot showing the distribution of GC composition. The mean GC composition of euchromatic gaps, non-euchromatic gaps, and sample-sourced assemblies (dashed vertical lines colored by discovery class), together with sample-sourced reference (black dashed vertical line). (B) Same information as panel A excluding repeats and repeat content annotated by RepeatMasker. (C) Violin plots showing the distribution of LINE, SINE, LTR, simple repeats, DNA elements, and satellite in non-redundant gap-closing sequences, and in randomly sampled sequences from GRCh38.

Genomic regions enriched in GC content are notoriously difficult to clone and sequence (Audano *et al.* 2019), while at the same time they may have significant biological effect (D’Onofrio *et al.* 1991). Thus, we examined the GC composition of the discovered gap-closing sequences. By visual examination, the distributions of GC content in both euchromatic gap-closing sequences and non-euchromatic gap-closing sequences are significantly different from the distribution of GC content of sampled sequences of human reference genome (Kolmogorov-Smirnov test *P* < 0.01). Both distributions were skewed toward lower GC content with a noticeable enrichment for < 30% GC (Figure 4A). The mean GC composition in euchromatic gap-closing sequences was significantly higher (*P* < 0.01, Wilcoxon test).

We further evaluated the repeat elements of the non-redundant gap-closing sequences and found that simple repeats and satellite sequences were significantly enriched within the gap-closing sequences when compared with randomly sampled sequences in the human reference genome (*P* < 0.001; Figure 4B). Especially, there was an enrichment of the proportion of satellites above 70% in non-euchromatic gap-closing sequences, which was not as obvious in euchromatic gap-closing sequences. After excluding all classes of repeat sequences, it became evident that both GC distributions (euchromatic and non-euchromatic) of gap-closing sequences follow the distribution of GC content in the human reference genome more closely (Figure 4C). Moreover, promoter regions associated with CpG islands in non-repeats sequences predicted by CpGProD (Ponger and Mouchiroud 2002) were also found in some of the non-redundant gap-closing sequences (Figure S3).

Although the functional significance of nearly all of these gap-closing sequences is largely unknown, 16 gaps fall within 17 distinct genes including 11 protein-coding genes (*AC142391.1*, *ECSCR*, *FAM20C*, *SHANK2*, *C1R*, *RILPL1*, *GALNT9*, *DLGAP4*, *CCNB3*, *CAPN8*, and *TWIST2*) as annotated by GENCODE (Frankish *et al.* 2019). To further explore the potential biological function in the gap-closing sequences, ORF Finder (Stothard 2000) was used to search for open reading frames (ORFs). The ORF Finder output contained 4,703 unnamed protein products, involving 166 gap-closing sequences for 91 gaps. In addition, analysis of the 4,703 non-redundant protein queries by aligning them to the BLAST non-redundant protein sequences (NR) database (ftp://ftp.ncbi.nlm.nih.gov/blast/db) with blastp (Altschul *et al.* 1990) (E value =1E-5) resulted in 1,571 hits. The majority (1,237, 78.7%) of the hits were hypothetical or predicted proteins that have not been well studied. Within the set of remaining hits (334), the most abundant hits (99) were matched to Histone acetyl-transferase MYST3, which includes zinc finger motifs that are known to be structurally diverse (Sri Krishna *et al.* 2003).

## Discussion

In this study, we used the assembly-to-assembly method to explore the gaps in the human reference genome. In total, we closed 132 genomic gaps and added 2.2 Mb NRS. A number of these gap-closing sequences showed a population-specific pattern and potential biological function. To our knowledge, this is the first time a systematical identification and characterization the gap-closing sequences in multiple individual genomes was performed. The key advantages of our study are: 1) the utilization of 17 distinct genome assemblies instead of a single assembly and 2) focus on both euchromatic and non-euchromatic regions.

Gaps in the reference genome can be grouped into two classes based on location: euchromatic region gaps and non-euchromatic region gaps. Previously studies predominantly focused on discovering gap-closing sequences in euchromatic regions (Chaisson *et al.* 2015; Pendleton *et al.* 2015; Seo *et al.* 2016). In the past few years, although there is two order of magnitude gain of read length delivered by third generation sequencing platforms which empowers resolution of many genomic regions with increased complexity, the read length is still not long enough to span centromeric and pericentromeric regions. Thus, investigators are still unable to accurately map and assemble reads to most of the heterochromatin and immediate sub-telomeric regions of the genome (Chaisson *et al.* 2015). This is perhaps the reason why euchromatic regions have received more research attention. However, with the further development of complementary technologies, the first gapless, telomere-to-telomere assembly of the human chromosome X was presented (Miga *et al.* 2019), which demonstrated that the completion of the human genome is now within reach. Efforts to complete the remaining human non-euchromatic gaps are desirable. Thus, in our work, we were not focused on euchromatic gaps only. Overall, 33.0% (63 of 191) euchromatic gaps and 11.7% (69 of 592) of non-euchromatic were closed. This finding is not surprising, considering the more complex nature of the non-euchromatic regions. When we question the robustness of the set of non-euchromatic region gap-closing sequences, we noticed that their polymorphic percentage (23.1%) is less than the percentage for the euchromatic region gaps (38.1%). The closed non-euchromatic gaps were mostly lying within model sequences or adjacent to these modeled regions, and those classified as telomere, heterochromatin, or short arm, so we speculate these regions are not as complex and repeats-rich.

Since the current human reference genome derived primarily from a single individual (Green *et al.* 2010), instead of a linear representation of a single haplotype, pan genomes of capturing the NRS have been developed to stand for the complex of human genome (Sherman and Salzberg 2020). Our alignment analysis revealed that the number of discovered NRS which overlap with NRS_Li (Li *et al.* 2019) or MSVs (Audano *et al.* 2019) from long-read *de novo* genomes is higher than the number of discovered NRS which overlap with the APG contigs (Sherman *et al.* 2019) from second-generation sequencing platforms. Compared with short-reads sequencing, long-reads sequencing technologies can reveal complex genomic regions such as regions that contain tandem and interspersed repeats, and varying GC content. This enables studies that can further drive genomic research and discovery (Goodwin *et al.* 2016). In addition, 18 gaps were identified to be covered by 13 sequences from NRS_Li (Audano *et al.* 2019). The number of gaps (18) is less than the number we filled (132) with similar assembly-to-assembly method. These results underscore the fact that many unexplored areas still remain and discovering gap-closing sequences will continue be an attractive and profitable area of genomic research. Overall, our findings are greatly beneficial in expanding the catalog of human NRS and facilitate the completion of the human reference genome, which is expected to contribute to association and functional analyses (Langley *et al.* 2018; Mefford and Eichler 2009), chromosome function study (Schueler *et al.* 2001), and human disease research (Eichler *et al.* 2010).

Although more than ten assemblies were used to close the gaps, only less than 17% of reference genome gaps could be closed in this study. Furthermore, the median length of gap-closing sequences (300 bp) is less than that of all unresolved gaps of the human reference genome (998 bp). This fact suggests that the majority of long gaps still remain unsolved. The advance of future sequencing technology and analysis methods will eventually solve this problem; an example of such breakthrough development is the first gapless, telomere-to-telomere assembly of a human chromosome X (Miga *et al.* 2019).
